# 

**Published:** 1894-07-07

**Authors:** 


**Notices of Births, Deaths, and Marriages** are inserted in this column at a charge of 2s. 6d. for an announcement not exceeding four lines.

**Notice to Advertisers and Subscribers.**

All Advertisements, Orders for Copies of The Hospital, and Business Communications should be addressed to **The Publisher, NOT TO THE EDITOR,** The Hospital, 428, Strand, W.C.

The Cost of Subscription, post free, is as follows:—Per week, 2½d.; per quarter, 2s. 8d.; per half-year, 5s. 3d.; per annum, 10s. 6d. Foreign:—Per Annum, 12s. 8d.; payable, in all cases, in advance.

### NOTICE TO CORRESPONDENTS.

All MS., Letters, Books for Review, and other matters intended for the Editor should be addressed The Editor, The Lodge, Porchester Square, London, W.

The Editor cannot undertake to return rejected MS., even when accompanied by stamped directed envelope.

**"Burdett's Hospital Annual," 1894.**

Fifth year of publication. 900 pp. Boards, gilt lettering. A most complete guide to British, Colonial, Indian, and American Hospitals, Dispensaries, Nursing and Convalescent Institutions, and Asylums. Price 5s. Published by The Scientific Press (Limited), 428, Strand, W.C.

**NOTICE.—The Index for Vol. XV. (ending March 31st, 1894) is on sale at the Office of this Journal, price 2½d., post free.**

### SCRAPS AND GLEANINGS.

The report of the Glasgow Hospital for Skin Diseases shows a balance in hand of £700 at the end of the year's working.

A munificent gift of £1,000 has been made by Mr. William Birke, of Welham Hall, Retford, to the Beckett Hospital, Barnsley.

A memorial window has just been erected at the Bristol Royal Infirmary, as a thank-offering " of one who has suffered and recovered."

An anonymous friend has presented £25 to the Aberdeen Royal Infirmary Corporation, and £25 to the charitable fund in connection with the asylum.

A house to house collection, as also an open air, was made last week at Londonderry, in furtherance of the much needed funds for the Eye and Ear Hospital of that locality.

The Friendly Societies of Stourbridge and the district held a special service recently with a view of assisting the funds of the Corbett Hospital. The collections amounted to £17.

The East London Hospital for Children, Shadwell, E., has received donations of £5 from the Carpenters' Company, and £100 from the trustees of Smith's (Kensington Estate) Charity.

A number of wealthy New York women have purchased a large tract of land at the foot of the Saranac Mountains, near Liberty, New York, and intend to create thereupon a Charity Hospital for Consumption.

A new wing to the New Brighton Convalescent Home (Liverpool) for women and children has been formally opened by the Countess of Grosvenor. One of the wards has been furnished by Lady Mary Fielder.

In answer to the appeal of the Board of Management of the British Home for Incurables for £5,000, to enable them to open the new buildings free of debt, the Worshipful Company of Leathersellers have sent a donation of £100.

An effort is being made to raise a special maintenance fund of £10,000 for the Alexandra Hospital for Sick Children with Hip Disease. The branch in connection with this institution at Bournemouth has had to be losed for want of funds.

A permanent isolation hospital is to be built for Heath Town (Wolverhampton). The intended hospital will be of corrugated iron, lined with match boarding, and the total cost, including appointments, will be at the moderate sum of £250.

The annual meeting in connection with the Balsall Heath Branch of the Birmingham Provident Dispensary was held recently. From the balance-sheet accompanying the report, it appears that the expenses are kept fairly well under the receipts.

The Yorkshire Home for Incurables has just celebrated its annual meeting. The number of inmates in the house during last year was 31, and the amount received from them was £380. The finances of the institution are in a satisfactory condition.

An application was recently made to the authorities of the Dundee Infirmary to provide instruction for female students. This request was not acceded to, since it would require greater ward space to carry out clinical instruction for male and female students.

The annual general meeting of subscribers to the Marlow Cottage Hospital was held recently. An unsatisfactory difference was pointed as existing between the receipts and the expenditure. Forty-one patients have been admitted to the hospital during the year.

There appears to be a growing necessity for some extension to the Queen's Hospital, Birmingham, as well as the General Hospital, and as such a state of things is unsatisfactory to the patients, a scheme of raising money to build a new wing is being mooted.

The hon. secretary of the Paddington Green Children's Hospital announces having received £800 in donations towards the building fund, and in view of the contract for the new hospital, which is announced to be completed at the end of the year at a cost of £11,000.

The Scottish football league, Glasgow, have intimated their intention of forwarding a contribution of £45 to the Edinburgh Royal Infirmary. A legacy free of duty has been left to the same institution from the trustees of the late Mr. George Fullarton, Dumfriesshire.

The committee appointed by the governors of the Newcastle Royal Infirmary are to meet the Town Moor authorities this month in reference to the site for the new building. It is much hoped that a grant of land the north side of the Qeazes Park will be given for this purpose.

In answer to the appeal of the Board of Management of the British Home for Incurables for £5,000, to enable them to open the new building free of debt, the Worshipful Company of Skinners have sent a donation of £25, and the Worshipful Company of Leathersellers £100.

The managers of the Royal Infirmary, Edinburgh, are again appealing for an increased subscription list. They are now calling the attention of the public to the extension of the premises, which, whilst giving a further accommodation, proves a very considerable drag on the resources of this national institution.

The forty-seventh annual meeting of governors of the Kent County Ophthalmic Hospital took place recently, the report of which institute stated that, owing to the increase in the number of in-patients, the coct of maintenance of the hospital has slightly increased, the amount in the year being £1,449, as against £1,401 in 1892.

With a view to increasing the funds being now raised towards the erection of the Gravesend Hospital, a Conversazione was held at the New Public Hall, a gathering which was attended with much success, the President, the Earl of Darnley, being present. A sum of £6,500 is required, towards which £1,300 has already been forthcoming.

Lady Jeune held recently at 79, Harley Street, a drawing-room meeting in aid of the North-Eastern Hospital for Children, Hackney Road. The hospital is in want of £7,000, of which £4,500 is for debt. The institution is, moreover, unable to accommodate infantile diseases in its immediate neighbourhood, and it is proposed to raise funds for its enlargement.

The annual report of the Dundee Infirmary, showed 2,229 patients registered on the books. The ordinary income showed an increase of £50 over that of last year, the annual subscriptions remaining unchanged. A handsome addition to the revenue of the institution has been supplied by Miss Harris, of Fernbank, who has transferred railway stock to the value of £3,000 for the hospital accounts.

### The Student of Medicine.

### APPOINTMENTS.

T. Bell, M.D., B.A.Dub., M.B., B.Ch., appointed Medical Officer of the Warrenpoint Dispensary District. F. C. Constant, L.D.S.R.C.S.Eng., appointed Dental House Surgeon to Guy's Hospital. J. Garner, L.R.C.P., L.R.C.S.Edin., L.F.P. and S. Glas., appointed Registrar and Assistant to the Surgeon of the Down County Infirmary, Downpatrick. A. Harnett, L.R.C.P., L.R.C.S.Edin., reappointed Medical Officer of Health to the Eastleigh Local Board. A. G. Haydon, M.D., M.R.C.S., L.R.C.P.Lond., appointed Physician in Charge of the Electric Department of the National Hospital for Paralysis and Diseases of the Heart, Soho Square, W. W. G. Holloway, M.D., B.A. Cantab., M.R.C.S.Eng., appointed Assistant Surgeon to the Central London Throat and Ear Hospital, Gray's Inn Road. W. S. Mackenzie, L.R.C.P., L.R.C.S.Edin., reappointed Medical Officer of Health to the Normanton Local Board. T. I. Mills, M.R.C.S.Eng., L.R.C.P.Lond., appointed Third House Surgeon to the Huddersfield Infirmary. W. Pogson, F.R.C.S.Eng., reappointed Medical Officer of Health for the Leeds Rural Sanitary Authority. C. S. Storrs, B.A., M.B., B.C.Cantab, appointed House Physican to the North-Eastern Hospital for Children, Hackney Road, N.E.

### VACANCIES.

The following vacancies are announced this week, the amount of salary in each case being given after the name of the institution:

### Resident Medical Officers.

Brighton, Hove, and Preston Dispensary.—House Surgeon for the parent establishment. Salary £140 per annum, with furnished apartments, coal, gas, and attendance, but no board. Applications and testimonials to J. W. Stride, Assistant Secretary, before July 10th.

East London Hospital for Children and Dispensary for Women, Glamis Road, Shadwell, E.—Resident Medical Officer. Must be registered practitioner in medicine and surgery. Salary £80 per annum, with board and residence. Applications and testimonials to Thomas Hayes, Secretary, by July 23rd.

North-Eastern Hospital for Children, Hackney Road, N.E.—Junior House Physician. For six months, commencing August 1st. Applications and testimonials to T. Glenton-Kerr, Secretary, 27, Clement's Lane, E.C., by July 12th.

### THE STUDENT OF MEDICINE—*continued.*

North Staffordshire Infirmary and Eye Hospital, Hartshill, Stoke-upon-Trent.—House Physician. Salary £100 per annum, increasing £10 per annum at the discretion of the committee, with furnished apartments, board, and washing. Applications to the Secretary by July 23rd.

Queen Charlotte's Hospital, Marylebone Road, N.W.—Resident Medical Officer. Appointment for four months. Salary at the rate of £60 per annum, with board and residence. Applications and testimonials to Arthur Watts by July 7th.

West Derby Union.—Resident Assistant Medical Officer at Mill Road Infirmary. Salary £100 per annum, with rations. Applications and testimonials to Harris P. Cleaver, Clerk to the Guardians, by July 10th.

West Norfolk Hospital, King's Lynn.—House Surgeon, who will also act as Secretary to the Weekly Board. Salary £80, rising £10 annually to £100, with board, residence, and washing. Applications and copies of testimonials to S. R. Lister, Secretary, by July 7th.

West Riding Asylum, Wakefield.—Two Resident Clinical Assistants. Board, furnished apartments, and attendance provided, but no salary. Appointment for six months. Applications to the Medical Director.

### Non-Resident Medical Officers.

Dental Hospital of London, Leicester Square.—Anæsthetist. Must be duly registered medical practitioner. Applications and testimonials to Francis Pink, Secretary, before July 9th.

London Hospital Medical College, Mile End.—Senior Demonstrator of Physiology and Lecturer on Biology. Salary for the former £150 per annum, and a proportion of fees paid for classes; and for the latter £100 per annum and class fees. Applications to Munro Scott, Warden, by July 7th.

London Throat Hospital, 204, Great Portland Street, W.—Secretary. Salary £55 per annum. Applications to the Chairman, Executive Committee.

North London Consumption Hospital.—Clinical Clerks, both for In-patient and Out-patient Departments. Applications to the Secretary, Lionel F. Hill, M.A., 41, Fitzroy Square, W.

University College, Dundee, St. Andrews University.—Professor of Chemistry. Applications to R. N. Kerr, Secretary, by July 7th.

Westminster Hospital, Broad Sanctuary.—Second Dental Surgeon. Candidates must attend the House Committee on Tuesday, July 10th, with testimonials.

Westminster Hospital Medical School.—Demonstrator of Anatomy. Applications to Mr. Spencer, the Dean, before July 10th.

### PASS LIST.

#### Society of Apothecaries of London.

The following gentlemen were appointed examiners, on Tuesday, June 19th, for the year 1894-95: J. C. Thorowgood, M.D.Lond., F.R.C.P. Lond.; F. Warner, M.D.Lond., F.R.C.P.Lond.; A. H. N. Lewers, M.D.Lond., M.R.C.P.Lond.; H. R. Crocker, M.D.Lond., F.R.C.P.Lond.; Sir H. R. Beevor, Bart., M.D.Lond., M.R.C.P.Lond.; A. P. Luff, M.D.Lond., M.R.C.P.Lond.; T. V. Dickinson, M.D.Lond., L.R.C.P. Lond.; C. Y. Biss, M.D.Cantab., F.R.C.P.Lond.; A. J. Richardson, M.D.Cantab., M.R.C.P.Lond.; C. B. Lockwood, F.R.C.S.Eng.; F. G Parsons, F.R.C.S.Eng.; P. T. B. Beale, F.R.C.S.Eng.; E. Cautley, M.D.Cantab., M.R.C.P.Lond.; H. F. Morley, D.Sc.Lond.; F. J. M. Page, B.Sc.Lond.; James Galloway, M.D.Aberd., M.R.C.P.Lond.; Harrington Sainsbury, M.D.Lond., F.R.C.P.Lond.; Andrew Clark, F.R.C.S.Eng.; W. A. Lane, F.R.C.S.Eng.; W. A. Frost, F.R.C.S.Eng.; A. T. Norton, F.R.C.S.Eng.; B. Pitts, F.R.C.S.Eng.; and B. Pollard, F.R.C.S.Eng.

The following candidates passed in

Surgery.—T. S. Biggs, Guy's Hospital; F. L. Blenkinsop, University College; M. S. Loewenthal, Wurzburg; C. A. Marrett, Charing Cross Hospital; J. P. Rerrie, New York, Bellevue; and O. O. Williams, London Hospital.

Medicine, Forensic Medicine, and Midwifery.—F. L. Blenkinsop, University College; E. Gill, St. Bartholomew's Hospital; R. H. Hayes, Guy's Hospital; M. S. Loewenthal, Wurzburg; M. H. C. Palmer, London Hospital; W. H. Richards, London Hospital; and O. O. Williams, London Hospital.

Medicine and Forensic Medicine.—F. A. M. Flegg, St. Thomas's Hospital, and H. J. L. Wales, Guy's Hospital.

Medicine.—E. A. Tudman, University College.

Forensic Medicine and Midwifery.—H. Roberts, St. Mary's Hospital, and A. L. Saunders, St. Bartholomew's Hospital.

Forensic Medicine.—E. H. Le G. Best, St. Bartholomew's Hospital; F. Clarke, St. Bartholomew's Hospital; G. G. B. Hein, St. Thomas's Hospital; and J. M. Ritchie, Birmingham.

Midwifery.—E. B. Barber, London Hospital; W. D. Macdonald, Guy's Hospital; E. Ransome, Guy's Hospital; D. F. Roberts, Guy's Hospital; and A. P. Woolright, St. Bartholomew's Hospital.

To Messrs. Blenkinsop, Clarke, Gill, Loewenthal, Marrett, Palmer, Rerrie, Richards, and O. O. Williams was granted the diploma of the Society entitling them to practice Medicine, Surgery, and Midwifery.

### The Hospital Advertiser.

### Medical Appointments.

**NORTH-EASTERN HOSPITAL for CHILDREN,**
HACKNEY ROAD, N.E.

JUNIOR HOUSE PHYSICIAN REQUIRED for six months, commencing on August 1st. Candidates must possess a medical and surgical qualification.

No salary, but Board and Lodging (including Washing) will be provided.

Applications, together with copies of not more than four testimonials must be sent to the Secretary at 27, Clements Lane, E.C., not later than July 12th.

T. GLENTON-KERR, Secretary.

27th June, 1894. (39)

**WEST RIDING ASYLUM, WAKEFIELD.**

